# Noninvasive Metabolic Imaging of Engineered 3D Human Adipose Tissue in a Perfusion Bioreactor

**DOI:** 10.1371/journal.pone.0055696

**Published:** 2013-02-06

**Authors:** Andrew Ward, Kyle P. Quinn, Evangelia Bellas, Irene Georgakoudi, David L. Kaplan

**Affiliations:** Department of Biomedical Engineering, Tufts University, Medford, Massachusetts, United States of America; Johns Hopkins University, United States of America

## Abstract

The efficacy and economy of most *in vitro* human models used in research is limited by the lack of a physiologically-relevant three-dimensional perfused environment and the inability to noninvasively quantify the structural and biochemical characteristics of the tissue. The goal of this project was to develop a perfusion bioreactor system compatible with two-photon imaging to noninvasively assess tissue engineered human adipose tissue structure and function *in vitro*. Three-dimensional (3D) vascularized human adipose tissues were engineered *in vitro*, before being introduced to a perfusion environment and tracked over time by automated quantification of endogenous markers of metabolism using two-photon excited fluorescence (TPEF). Depth-resolved image stacks were analyzed for redox ratio metabolic profiling and compared to prior analyses performed on 3D engineered adipose tissue in static culture. Traditional assessments with H&E staining were used to qualitatively measure extracellular matrix generation and cell density with respect to location within the tissue. The distribution of cells within the tissue and average cellular redox ratios were different between static and perfusion cultures, while the trends of decreased redox ratio and increased cellular proliferation with time in both static and perfusion cultures were similar. These results establish a basis for noninvasive optical tracking of tissue structure and function *in vitro*, which can be applied to future studies to assess tissue development or drug toxicity screening and disease progression.

## Introduction

Two-dimensional (2D) cell culture systems and animal models are routinely used to assess toxicity for drug screening and to gain insight into diseases. Although these systems are useful, there is growing recognition of current limitations with 2D cell culture and rodent models, as they have not provided consistency in correlations to human clinical outcomes [1], leaving room for new options to fill this void. For example, animal models are utilized for many studies; however, when assessed for complex disease treatments including but not limited to treatments for viral (HIV), multibacillary (leprosy), and respiratory diseases, they usually fail to adequately represent how clinical treatments affect humans [2], [3], [4]. This is due to the complex differences in immunological responses that humans and other animals exhibit compared to one another, as well as differences in metabolic and physiological responses. Other complications arise when using 2D cell cultures as human models for clinical applications, which are inherently caused by the absence of multi-dimensional inputs and complex human tissue transport and signaling. Tissue engineering has emerged over the last two decades as an alternative to address the limitations of animal models and 2D cell cultures in modeling the biochemical and structural characteristics of native three-dimensional (3D) human tissues. The ability to combine cells and matrices to generate 3D human tissues in bioreactors has advanced rapidly in recent years to make tissue engineered constructs utilizing human cell sources a more advantageous model for studies relevant to clinical applications.

However, significant challenges exist in imaging and characterizing 3D tissues. Current standard approaches, such as histology and quantitative polymerase chain reaction (qPCR) techniques, are inherently destructive and cannot be used to continuously monitor tissue changes over time. Cells cultured in 2D are more easily observed for cellular density and localization over time through noninvasive means because their entire surface is easily observable on a plate. In contrast, analysis of 3D tissue cultures is often limited by the difficulty of visualizing cell features without mechanically sectioning the culture. By sacrificing each 3D culture to assess its biochemical or structural characteristics, dynamic spatiotemporal changes associated with tissue development or drug treatments cannot be measured. Further, the costs and complexity associated with such assessments limit their utility, and substantially more samples are needed to provide conclusive data over the same number of time points when compared to non-destructive analyses.

Different optical imaging methods capable of noninvasively tracking metabolic and morphological changes in 3D tissues over time include confocal microscopy, two-photon excitation fluorescence (TPEF), and fluorescence lifetime imaging, all of which can analyze morphological and biochemical features of tissues [5], [6], [7]. Recently, significant advances in optical imaging methods capable of probing endogenous cellular components in 3D specimens have emerged as a new approach to the challenges of monitoring tissue characteristics over time, obviating the need for destructive analysis [8], [9], [10]. Metabolic activity and cellular differentiation status can be assessed by isolating different metabolic fluorophores as endogenous sources of contrast. Specifically, the cofactors NAD(P)H and FAD, which link the tricarboxylic acid cycle to oxidative phosphorylation, can be isolated and quantified to assess osteogenic or adipogenic differentiation through TPEF imaging of human mesenchymal stem cells [9], [11]. Quantifying the relative fluroescence of NAD(P)H and FAD, provides a measure of the balance between energy storage (e.g. triglyceride synthesis) and consumption, which changes during processes such as adipogenic differentiation. These methods capable of non-destructive, live cell imaging within 3D human tissues are particularly amenable to drug and toxicology screening applications that require repeated dynamic measurements of cell function.

An aim of this study was to understand metabolic changes in engineered 3D human adipose tissue using a TPEF imaging platform [8]. TPEF imaging provides an ideal approach to localize specific fluorescent biomolecules within a 3D tissue. The use of near infrared light enables deeper penetration into the tissue constructs, and the simultaneous absorption of two photons provides intrinsic depth sectioning without the need for confocal detection using a pinhole. As a result, TPEF imaging can be used to detect the presence of metabolic cofactors such as FAD and NAD(P)H with minimal risk of photo-damage. By measuring the relative fluorescent concentrations of these two cofactors with a high sensitivity, insights have been gained into both anabolic and catabolic behavior of biological systems *in vitro* [9], [11], [12]. The expression of these cofactors is known to change during drug treatment, differentiation, and cell division, thus providing a sensitive indicator of cellular metabolic behavior [13], [14]. Quantifying cellular differentiation, proliferation, and drug responses is paramount in elucidating clinical treatments for injuries, pathogenesis, and toxicity. Unlike mass spectroscopy or enzyme-linked immunosorbent assays (ELISA), TPEF-based metabolic analysis can detect both temporal and spatial changes in cell responses to injury, disease, or drug treatment.

Modern bioreactor systems allow for more physiologically relevant models of human tissue compared to more traditional static tissue culture plates. Bioreactor systems can be designed to accommodate perfusion flow, media collection, and noninvasive imaging by designing them with imaging windows compatible with fluorescence imaging modalities. These imaging interfaces also have the potential for repeated TPEF imaging as a noninvasive approach to assess the structure of biomaterials, formation and deposition of new extracellular matrix, cellular viability, and metabolic cofactors of *in vitro* tissue systems. The goal of the present study was to implement a perfusion bioreactor system interfaced with noninvasive optical imaging tools to track the metabolic function of engineered 3D adipose tissue. The development and validation of such a bioreactor system would provide a powerful drug screening platform that enables a non-destructive quantitative measure of lipid metabolism in the study of diseases such as obesity and diabetes. To achieve such goals, metabolic monitoring in the bioreactor system was compared with static 3D cultures, and these outcomes were evaluated and confirmed against more traditional histological assessments of the tissue.

## Materials and Methods

### Ethics Statement

The protocols for engineering 3D vascularized human adipose tissue in vitro have been described previously by our lab [15]. hASCs were acquired from subcutaneous adipose tissue which was obtained from abdominoplasties approved under Tufts University IRB (Tufts University IRB Protocol #0906007) from Tufts Medical Center, Department of Plastic Surgery. Since the tissue was taken for an elective surgery and would have been routinely discarded and no identifying information about the patient was obtained, only verbal consent from the patient was required. HUVECs were obtained from Lonza (catalog #CC-2519, HUVEC-Umbil Vein, pooled donor cells, EGM,cryo amp). Verbal consent was approved by the Tufts University, Medford Campus Institutional Review Board (IRB). The patient was asked to review an informational sheet describing what researchers would do with their otherwise discarded tissue. Only if assent was collected, the researchers would be contacted after surgery by the medical staff to pick up the de-identified tissue. Verbal consent was deemed appropriate since no patient identifiers were collected and because the patients had undergone elective surgeries where the tissue of interest would have been discarded. Patients had undergone elective weight loss surgeries, the tissues resulting from these surgeries are routinely discarded. Since we were only using tissue that was procured for other purposes and would be otherwise discarded our IRB committee deemed full written consent unnecessary. Prior to surgery, patients were asked to review a patient informational sheet explaining how researchers would plan to use their discarded tissue. The sheet clearly stated that they may refuse to allow their discarded tissue to be used without any effect to their medical care. If the patient consented, the otherwise discarded tissue would be set aside for research. Additionally, no patient identifiers were collected and therefore all samples were de-identified. Patients were asked to review the informational sheet (as described above). If they consented verbally, a nurse would called the researchers, once the surgery was over and everything went well, to pick up the de-identified tissue. An assent log is kept of dates and initials of the researcher on the IRB protocol who picked up the tissue.

### Bioreactor

The 3DKUBE™ system (Kiyatec, Pendleton, South Carolina) was used in the present study with some modifications. The chamber accommodates a segregated culture of two samples, is compatible with unidirectional perfusion flow, and contains an imaging interface for each sample. The 3DKUBE™ chamber accommodates a 6.0 mm diameter×8.8 mm height internal volume for tissue placement. An external media reservoir was used to sustain the 3D tissue and provide for perfusion flow supply. The barrel of a 30 mL syringe with the plunger removed was used for this purpose, and the top of the syringe was plugged using a Size 3 VWR Black Rubber Stopper (Radnor, PA). In order to supply oxygen and remove carbon dioxide from the system, an outlet for gas exchange was implemented using a Millex-FG, 0.22 µm, hydrophobic PTFE, 25 mm diameter, EtO sterilized air filter. The gas exchange port was put at the site of the black rubber stopper at the top of the media reservoir. The chamber, peristaltic pump, and media reservoir for gas exchange were connected in series via platinum cured silicone and PharMed tubing (Cole Parmer, Vernon Hills, IL) using luer lock to barb connections. Each component used in the bioreactor system was sterilized by autoclaving with the exception of the modified 3DKUBE™ chambers and media reservoir (syringe barrel/rubber stopper), which were sterilized using a 24 hour EtO sterilization cycle. For each tissue sample introduced to the bioreactor system there was one 3DKUBE™, one 30 mL syringe barrel, one perfusion pump cassette, and approximately 18″ of combined tubing.

**Figure 1:**
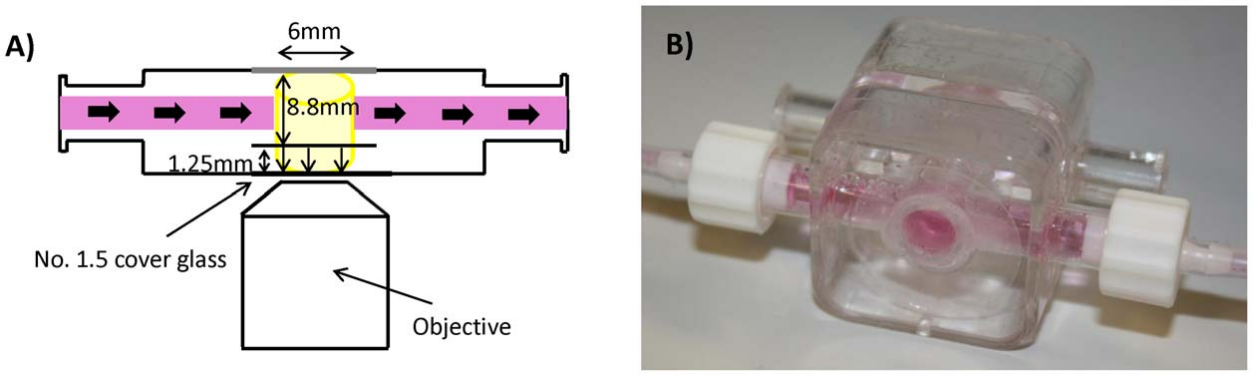
Bioreactor Design. A) Modified 3DKUBE™ design where the original imaging interface is bored out to extend the depth of the chamber from 8.8 mm to 10.05 mm deep and replaced with No. 1.5 cover glass. This brings the tissue sample closer to the objective in order to accommodate a shorter working distance required for higher magnification levels. B) Photograph of the modified 3DKUBE™.

The original imaging interface for the 3DKUBE™ was a 1.25 mm thick polystyrene plastic window which was not compatible with the 63× objective (1.2NA, 220 µm working distance) used for TPEF imaging (Fig. 1). To accommodate imaging with this objective, the 3DKUBE™ polystyrene imaging interface was removed and replaced with No. 1.5 thickness (Fischer Scientific, Hampton, NH) cover glass. This modified imaging window increased the internal height of the chamber to 10 mm, and allowed for imaging up to a depth of 200 µm (Fig. 1B).

### Perfusion

Optimal flow rates for tissue engineering applications are generally on the order of 400–800 µm/s [16]. These flow rates have been utilized for cardiac and osteogenic tissue engineering, and correlates appropriately to native interstitial lateral flow velocities which have been measured at 500 µm/s [16], [17], [18], [19]. Using 10 rpm for the peristaltic pump and Masterflex PharMed tubing size #13 (0.03″ ID) an average flow rate of 0.0056875 mL/s was measured. Considering the cross-sectional area of the bioreactor chamber inflow and outflow ports, the lateral flow rate perfusing through the adipose tissue samples within the bioreactor chambers was 412.5 µm/s.

### Engineered 3D Human Adipose Tissue

The protocols for engineering 3D vascularized human adipose tissue *in vitro* have been described previously by our lab [15]. Porous silk scaffolds were used to house the cells and fit into the bioreactors. The benefits for the silk biomaterial are the mechanical durability and very slow degradation (months to years), along with biocompatibility and success in 3D adipose tissue formation in our prior studies [9], [15]. We have previously described in detail the preparation and characterization of these porous silk scaffolds for the system [20]. Briefly, the *Bombyx mori* silk cocoons were chopped and boiled in a 0.2M sodium carbonate solution, and rinsed three times in distilled water, and then dissolved in a 9.3M lithium bromide solution [21]. The salt was removed by dialysis against distilled water before being centrifuged to remove any particulates. An 8% w/v silk aqueous concentration was used to form the scaffolds, to which 4 g of sieved granular sodium chloride, particle size 500–600 microns, was added to 2 mL silk solution and allowed to solidify for 72 hrs at room temperature. The scaffolds formed were rinsed in distilled water for 48 hrs to leach out the salt particles. The final scaffolds were punched out into 6×10 mm (*diameter×height*) scaffolds. The scaffolds were autoclaved for sterilization prior to cell seeding.

HASCs were acquired from subcutaneous adipose tissue which was obtained from abdominoplasties approved under Tufts University IRB (Tufts University IRB Protocol #0906007) from Tufts Medical Center, Department of Plastic Surgery. Since the tissue was taken for an elective surgery and would have been routinely discarded and no identifying information about the patient was obtained, only verbal consent from the patient was required. HUVECs were obtained from Lonza (catalog #CC-2519, Walkersville, MD) and cultured according to the manufacturer's protocol in EGM complete media (Lonza, Walkersville, MD). Both human adipose derived stem cells (hASCs) and human umbilical vein endothelial cells (HUVECs) were grown to confluency in 2D cultures before being seeded onto porous silk protein scaffolds at specified cell densities and time points. HASCs were cultured in Dulbecco's Modified Eagle's Medium with Ham's F12 (DMEM/F12) supplemented with 10% fetal bovine serum (FBS) and 1% penicillin streptomycin fungizone (PSF). At 2 days post-confluence, the hASCs were switched to adipogenic induction media comprised of DMEM/F12, 3% FBS, 1% PSF, 500 µM 3-isobutyl-1-methylxanthine (IBMX), 5 µM 2,3-Thiazolidinediones (TZD), 1 µM dexamethasone, 17 µM pantothenate, 33 µM biotin and 1 µM insulin. Media supplements for hASC media were purchased from Sigma-Aldrich (St. Louis, MO), media, FBS, and PSF were purchased from Invitrogen (Carlsbad, CA).

The sterile scaffolds were soaked in EGM media to promote cell adhesion and media was aspirated after 1 hr. Trypsinized HUVECs were re-suspended at a concentration of 26 million cells per mL EGM, and seeded in three 20 µL aliquots. The seeded scaffolds were then incubated for 1 hr to allow for cell attachment before adding 6 mL of EGM media to the well. The same seeding protocol was followed for seeding hASCs, except the cell concentration used was 13 million cells per mL hASC maintenance media (same as adipogenic media without IBMX and TZD). Scaffolds seeded with both hASCs and HUVECs were maintained in a 1∶1 ratio of EGM media to adipogenic maintenance media.

### Timeline

**Figure 2:**
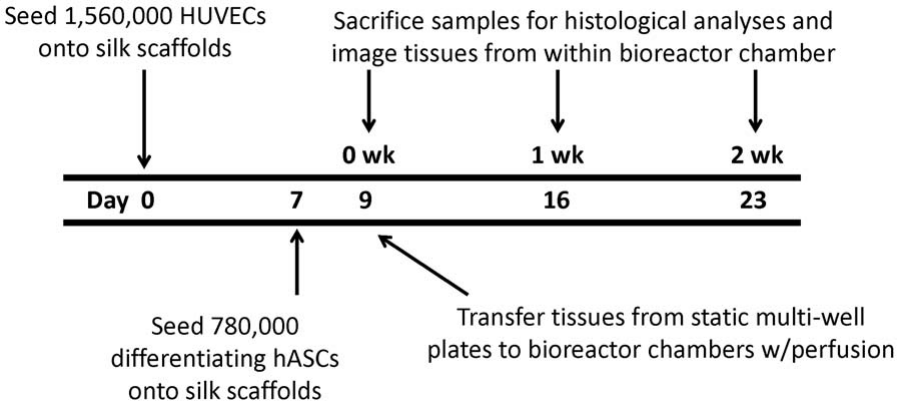
Experimental timeline describing cell seeding, induction and imaging time points. The total number of cells seeded on each scaffold was scaled up by scaffold volume to maintain a consistent density with previous work [15].

The experimental timeline is shown in Fig. 2, where Day 0 corresponds to when HUVECs are first seeded onto the silk scaffold. At Day 0, adipogenic differentiation of the confluent 2D hASC cultures is induced through the addition of soluble factors [9], [15], and on Day 7 the differentiating hASCs are seeded onto the scaffold. Because the tissues in this experiment are introduced to perfusion flow, initial cell adhesion to the silk scaffolds is important. To facilitate this step, the tissues were not introduced to the perfusion bioreactor system until Day 9 (two days after seeding the differentiating hASCs), in order to ensure that the cells remain anchored to the scaffold well after seeding. On Days 9, 16, and 23, the samples were imaged from within the 3DKUBE™ chamber through the custom fitted cover glass. In the data presented in the results, Days 9, 16, and 23 correspond to the time points 0 wk, 1 wk, and 2 wks

### Fluorescent Object Classification and Redox Ratio Analysis

Previous analysis of silk as a biomaterial in solution, gel, and scaffold forms found that the fluorescent properties of silk was dominated by tryptophan fluorescent contributions [22]. This factor results in peak single-photon and two-photon excitation wavelengths of roughly 280 and up to 700 nm, respectively [8]. The broad two-photon excitation spectrum of silk results in substantial silk fluorescence even at the longer excitation wavelengths used in this study. However, the relative ratios of silk fluorescence intensity within the specific excitation and emission channels used here are slightly different than the relative ratios of cellular fluorescent intensities that detect NAD(P)H and FAD. By utilizing linear discriminant analysis, the fluorescence intensities of silk and cells can be properly classified and differentiated from one another [9].

Adipose tissue samples were imaged with a confocal microscope (Leica TCS SPC2 confocal microscope, Mannheim, Germany) using a Ti:Sapphire tunable laser (Mai Tai; Spectra Physics; Mountain View, CA). The samples were held flush with the glass coverslip interface allowing a 200 µm penetration depth using a water immersion 63× objective. The fluorescence of NAD(P)H and FAD were isolated by exciting the samples at specific wavelengths and then gathering fluorescence intensities across two different ranges using non-descanned photomultiplier tubes (PMTs). NAD(P)H was excited using a 755 nm excitation wavelength, and the subsequent fluorescence intensity was measured from a PMT detector with a 460(±20) nm emission filter. FAD was similarly isolated with an excitation wavelength of 860 nm and PMT detector with a 525(±25) nm emission filter [9], [11]. A series of depth resolved image stacks were taken from 0–40 µm, 80–120 µm, and 160–200 µm at 2.5 µm increments. Each image volume was then run through a custom image processing program in Matlab that segmented 3D objects and utilized a linear discriminant analysis to distinguish between cells and the silk scaffold structure based on differences in fluorescence intensity at the different emission and excitation wavelengths [9].

**Figure 3:**
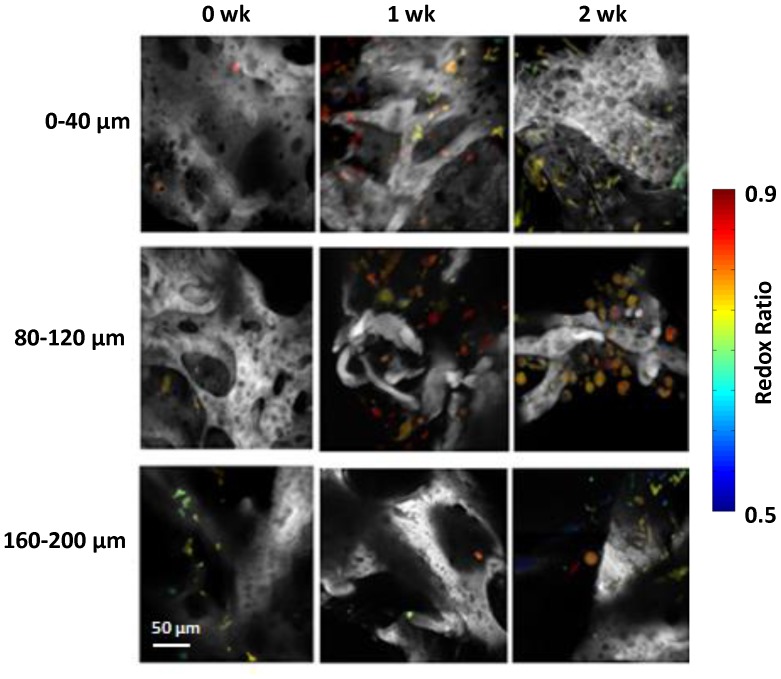
False colored redox maps of adipose tissues *in vitro* after 0, 1, and 2 wks within the perfusion environment. The grayscale structures in the images are the silk biomaterial 3D scaffolds, while the sparsely distributed colored objects are cells.

After identifying the cells within the 3D depth resolved images, the cell fluorescence intensities, redox state, cell morphology and size were quantified by software [9]. The 12-bit fluorescence intensity image at each excitation and emission combination was normalized by laser power and detector gains [9]. The average NAD(P)H and FAD fluorescence intensities were computed for each cell, and a cell-specific redox ratio of FAD/(NAD(P)H+FAD) was computed and expressed using a false color redox map (Fig. 3). The calculated redox ratio has been used to measure metabolic behavior, and the analysis performed here in a perfusion environment was compared to our previous research conducted in static cultures [9].

### Histology

To complement the redox maps and data acquired from the noninvasive imaging analyses, histological H&E stained images were imaged to gain an understanding of cellular proliferation, cellular localization, and ECM formation within the tissues. Tissues were fixed in formalin, and put through a series of dehydration steps and finally paraffin. Paraffin embedded tissues were then cut into 6 µm thick slices, and rehydrated and stained with H&E, according to standard histology protocols [23], [24]. Images of the H&E stained samples taken at the edge and the middle sections of the tissues using 10× and 40× objectives. The tissue ‘edges’ were defined as the surface of the tissue that was imaged through redox ratio analysis from the bottom side of the bioreactor chamber, while ‘middle’ images lie within the same circular cross sectional area of the perfusion inflow and outflow ports of the bioreactor chambers taken from halfway within the tissue.

### Statistical Analysis

A one-way analysis of variance (ANOVA) was performed to determine whether there was a significant difference between the mean redox ratios at the three different time points. Post-hoc two sample t-tests were performed to determine differences between the 0 wk and 1 wk time points, and between the 1 wk and 2 wk time points. Similar t-tests were performed to compare redox ratios between perfusion and previously published static cultures. All t-test statistics were Bonferroni-corrected and significance was defined when p<0.05.

## Results

**Figure 4:**
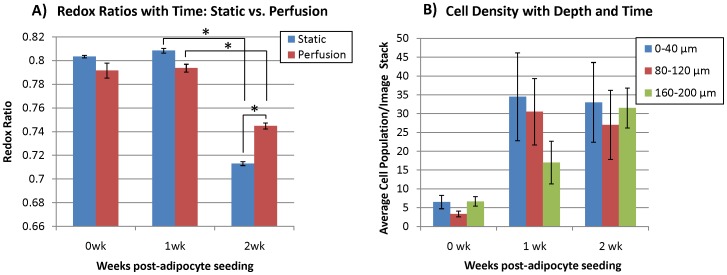
Changes in cell redox ratio and density with time. A) Average cellular redox ratio at 0, 1, and 2 wks in perfusion compared to previously published static culture experiments [9]. In both static and perfusion culture, no change in redox was observed between 0 and 1 wks, but the redox ratio decreased (p<0.001) from 1 to 2 wks. Although a significant decrease was observed in both cultures at wk 2, the redox ratio was signficantly higher in the perfusion cultures. B) Cell population measurements per image stack (238×238×40 µm volume) at 0, 1, and 2 wks in perfusion flow demonstrate an increase over time, but substantial sample-to-sample variability.

After the redox ratio calculations were performed on the images, the data obtained from the perfusion system was compared with data acquired from static cultures gathered in our previous study [9] (Fig. 3 & 4A). Neither culture showed a significant change in redox ratio between their respective 0 wk and 1 wk time points (Fig. 4a). At the 0 wk time point the sample mean redox ratio was 0.803±0.0021 for the static culture, and 0.792±0.0137 in the perfusion culture. At 1 wk, the static culture sample mean redox ratio was 0.808±0.040, and in perfusion the sample mean was 0.794±0.0066. However, both the static and perfusion systems showed a significant decrease in redox ratio from the 1 to 2 wk time points. From 1 to 2 wks, the average redox ratio of cells found within the static culture dropped from 0.808±0.040 to 0.713±0.0032 (p<0.001). In perfusion, the average cellular redox ratio from 1 to 2 wks significantly decreased (p<0.001) from 0.794±0.0066 to 0.744±.0049. In addition, the static and perfusion cultures at the 2 wk time point were significantly different (p<0.001) where the perfusion culture showed a higher redox ratio after 2 wks.

**Figure 5:**
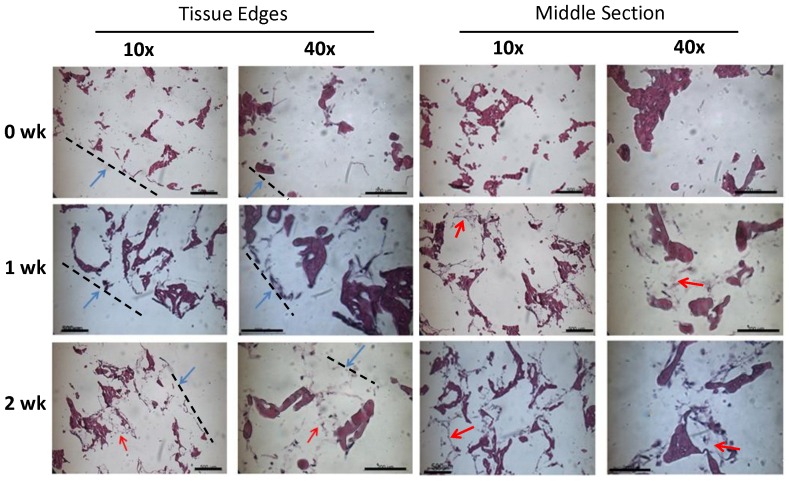
H&E stained images taken of the edges and middle of sacrificed adipose samples after 0, 1, and 2 wks in perfusion. **Images were acquired at 10× and 40×.** The dark purple structures are part of the silk scaffold, the lighter purple is ECM, and the dark spots within elongated structures are nuclei within cells. The blue arrows and dashed line specify the location of the edges of the tissues. The red arrows point out ECM formation in at the 2 wk time point. 10× scale bars are 500 µm. 40× scale bars are 200 µm.

The TPEF images were further analyzed to yield data on cellular density with respect to imaging depth. The relationship between cellularity with respect to depth at 0, 1, and 2 wks in perfusion is shown in Fig. 4B. The observed trend was that cellularity was most dense at the surface layers after 1 wk and then the cells gradually began to distribute themselves more evenly throughout the three imaging depths in perfusion. To supplement the cell count measurements made noninvasively, the cellularity within the silk scaffolds was also assessed as a function of time through histology. H&E images show qualitatively that there was no substantial difference in cellularity and ECM formation with respect to depth within the tissue samples at the end of end of test (2 wks) in perfusion culture (Fig. 5).

## Discussion

Over two weeks, the perfusion bioreactor system and imaging platform described here demonstrated the ability to obtain metabolic redox ratio data from 3D tissue engineered human adipose tissue samples *in vitro*. The average cellular redox ratios gathered through TPEF imaging and subsequent image processing showed similar trends as has been observed in static cultures; consistent redox ratios over the first week were followed by a significant drop at 2 wks. The decrease in redox ratio from 1 to 2 wks is consistent with previous work and indicates hASC differentiation [9]. Upon differentiation, hASCs are not expected to continue to proliferate. This expectation is supported in by the relationship between the average cellular redox ratios (Fig. 4A) and the average cellularity (Fig. 4B). There is no significant change in redox ratio observed within the first week of testing, which would suggest that hASC differentiation is low and proliferation is continuing. This is further supported by the increase in cellularity observed from the 0 to 1 wk time points. Then as the rate of differentiation begins to increase between the 1 and 2 wk time points, the redox ratio decreases and cellular proliferation halts, as observed in the more modest increase in cellularity from 1 to 2 wks compared to the rapid increase from 0 to 1 wks.

Previous analyses demonstrated a decrease in redox ratio coincided with the formation of lipid droplets [9]. The underlying mechanism driving the relationship between redox ratio and lipogenesis has been attributed to an accumulation of mitochondrial NADH during de novo fatty acid synthesis. Energy storage through fatty acid synthesis and lipid droplet formation likely only occurs when the relative energy consumption is low. Decreases in ATP production and NADH oxidation by the electron transport chain combined with an increase in the conversion of NAD+ to NADH by the pyruvate dehydrogenase complex when breaking down pyruvate for fatty acid synthesis collectively cause a decrease in the mitochondrial ratio of NAD+/NADH. Furthermore, FAD bound to lipoamide dehydrogenase in the pyruvate dehydrogenase complex will likely remain in the oxidized form when the less NAD+ is present to accept the electrons. Collectively, these mechanisms are thought to provide a consistent decrease in redox ratio during lipogenesis in both static and perfusion cultures.

The smaller decrease in redox ratio in the perfusion culture relative to static cultures could be a result of a slight delay or reduction in the number of hASCs differentiating and undergoing lipogenesis at 2 wks. Alternatively, the endothelial cells may be more viable or proliferative in the perfusion culture than in static cultures causing a dampening of the expected decrease in redox ratio associated with adipogenic differentation. However, previous comparisons between adipogenic differentiation in hASC monocultures and hASC-endothelial co-cultures yielded no differences between the redox ratio of the two groups beyond Day 1, suggesting endothelial cells likely have little effect on redox ratio changes over time. Nonetheless, CD-31 staining for endothelial cells in histological sections can be performed in future work to gain more insight into the immediate behavior of endothelial cells in the 3D cultures with respect to cellular proliferation and angiogenesis. It is also worth noting that experimental differences between the current perfusion study and previous static culture study [9], including a human endothelial cell source and the different solvents used during silk scaffold processing, may cause a small difference in the temporal pattern of differentiation. Finally, it is worth considering that the mechanical environment produced by perfusion may inhibit adipogenic differentiation or lipogenesis to some degree or selectively detach mature adipocytes with large lipid stores.

Previous studies involving adipose tissue engineering and subsequent characterization for clinical applications have set forth both protocols and analytical benchmarks by which adipose tissue can be analyzed *in vitro* and compared. Adipose tissue has been engineered using both synthetic and natural polymer scaffolds with both human mesenchymal and pluripotent stem cells [9], [15], [25], [26]. These studies have provided an understanding of how lipid droplet formation, cellular proliferation, lumen formation, and cell metabolism change over time. These observations demonstrate cellular distribution, ECM growth, and vascularity within engineered adipose tissues grown in static culture. Through these previous studies, a standard set of analytical tools used to evaluate the 3D engineered adipose tissue have been developed which include, but are not limited to, histology, immunohistochemistry, and two-photon imaging [7], [9], [27]. In this study histological analysis with H&E staining was performed and is capable of measuring this cellular distribution and ECM growth, which is helpful in supporting and verifying the data collected from TPEF imaging. In previous work, the relationships have been established between TPEF imaging and Oil Red O staining for lipid droplet formation, as well as CD31 staining for endothelial cell organization [9]. Future work with the bioreactor system used in the current study will further confirm that TPEF relationships with these other traditional analytical benchmarks are maintained in a perfusion environment.

As observed in Fig. 5, there is little, if any, ECM present in the 0 wk samples. This is likely because these samples had only been seeded with differentiating hASCs for two days, which is not sufficient time to accommodate ECM formation. There is, however, ECM present in the 1 and 2 wk cultures (note red arrows on Fig. 5). This is likely a result of increased cellularity within the tissues, accomplished through more allowed time for the cells to adhere to the silk scaffolds and generate an ECM. The ECM of adipose tissue primarily consists of a loose collagenous matrix to provide structural support. It would be expected that if these tissues had remained in culture for longer times, that the cellularity of the tissues would increase while a more robust and expansive ECM developed. We have previously reported such findings using more traditional destructive modes of imaging for engineered 3D human adipose tissue systems. ECM and cellularity have been visually observed through immunohistochemistry, Oil Red O, and image processing cell count algorithms [9], [15], [27].

Although substantial variability was observed in cell densities, there did appear to be a trend toward higher cell density in the superficial layer of the tissue at 0 wks compared to the deeper sections. This is not surprising because the cells were only just seeded, and most likely did not have much opportunity to migrate throughout the silk scaffolds. Aggregation of the cells was observed within the superficial (0–40 µm) range at the 1 wk time point within the perfusion cultures. However, by 2 wks, cellularity appeared to level out within each of the layers (0–40 µm, 80–120 µm. 160–200 µm) in both the noninvasive imaging analysis (Fig. 3) and H&E stains in the perfusion culture (Fig. 5). Longer experimental time points would be required to confirm this trend, but a more homogenous distribution of cells within a 3D scaffold in perfusion culture environment relative to static has been observed elsewhere [16].

Relative to normal tissue culture plate cell observations, the perfusion bioreactor and imaging system affords distinct advantages, including more physiologically relevant perfusion flow and the ability to perform repeated two-photon imaging analyses with submicron resolution. The impact of this approach is improved efficacy and relevance of future analytics by providing a platform for continuous metabolic and structural analysis of 3D human tissue samples. The approach also implicitly allows for improved cost and labor efficiency by offering an alternative to non-destructive analyses, saving substantial resources. Certainly, optical microscopy evaluation is limited to the outer 0.5–1 mm of the tissue. While no depth-dependent differences in redox ratio were observed in this or previous studies [9], additional evaluation of unstained tissue sections may provide further insight into the necessary scaffold and bioreactor design considerations for high-throughput drug and toxicity screening applications.

In conclusion, the research here demonstrates the ability to interface non-linear optical microscopy with 3D human tissue constructs within a perfusion environment for noninvasive continuous analysis. The perfusion bioreactor system was modified to be compatible with advanced imaging capabilities using high numerical aperture objectives, but was still capable of sustaining continuous flow over weeks to months with media replenishment. The perfusion bioreactor system sustained cultures over the span of weeks, and demonstrated potential to continue for months, as has been previously performed in static cultures. Multiple samples that entered the perfusion system were able to remain in a sterile environment and maintain viability throughout the experiment. TPEF imaging provided metabolic and cellular distribution information which correlated with standard histological analysis. These destructive and non-destructive imaging approaches supported each other, both showing adipose tissue formation as characterized by an increase in cellularity throughout testing, and ECM growth as shown through histology. A constant redox ratio was observed over the first week of testing in both the current perfusion study and previous static study [9], but both demonstrated a significant decrease in redox ratio from the 1 to 2 wk time points, suggesting lipogenesis. This combination of quantitative metabolic imaging and 3D tissue engineering using perfusion bioreactors represents a step toward low-cost, high-throughput methods to study more physiologically relevant engineered human tissue systems in applications such as disease models, drug screening, or developmental biology.
